# FEASIBILITY OF TRAINING CAREGIVERS ONLINE TO DELIVER LIFE REVIEW DEPRESSION INTERVENTION AT HOME

**DOI:** 10.1093/geroni/igae098.1442

**Published:** 2024-12-31

**Authors:** Christina Miyawaki, Angela McClellan, Erin Bouldin

**Affiliations:** University of Houston, Houston, Texas, United States; University of Houston, Houston, Texas, United States; University of Utah, Salt Lake City, Utah, United States

## Abstract

Due to the high prevalence of depressive symptoms and dementia in older Americans (≥65 years), we developed a six-week depression intervention, Caregiver-Provided Life Review (C-PLR) for care recipients (CRs) with early-stage dementia and mild depression and taught their family caregivers (CGs) life review skills online. The objective of this study was to examine the feasibility of training caregivers via an online video course and the impact of delivering the intervention at home (N=20 dyads). A mixed-methods design (surveys and interviews) was used for this study. We collected pre- and post-measures of CRs’ depression (primary outcome) and life satisfaction, CGs’ burden, rewards of caregiving, and dyads’ relationship quality (secondary outcomes) and compared them using paired t-tests. We took fidelity scores to measure CGs’ feasibility to deliver life reviews adhering to the intervention protocol. CGs were on average 64 years old, White (65%), married (65%), college-educated (70%), working or retired (40% each), and female (90%) in good/excellent health (100%). Their CRs were 80 years old (mean), White (70%), female (50%) in poor/fair health (50%). CGs’ average fidelity check-in score was 15.9±0.27 (out of 16), indicating excellent adherence. The C-PLR intervention significantly reduced CRs’ depressive symptoms (p=0.034) and increased CGs’ rewards of caregiving (p=0.019). Caregivers’ post-intervention qualitative interviews supported the quantitative results that online-trained caregivers could deliver the intervention by protocol without adding caregiver burden. This study demonstrated that the C-PLR, an innovative, cost-effective intervention with a flexible delivery schedule could make a positive impact on both caregivers’ and care recipients’ mental health.

